# GRP78 promotes rabies virus entry through interacting with viral receptors

**DOI:** 10.1128/jvi.00039-26

**Published:** 2026-04-14

**Authors:** Chong Wang, Zhiyuan Wen, Chen Wang, Qilong Liu, Hailin Zhang, Lei Shuai, Xijun Wang, Jinying Ge, Jinliang Wang, Zhigao Bu

**Affiliations:** 1 State Key Laboratory of Animal Disease Control, Harbin Veterinary Research Institute, Chinese Academy of Agricultural Sciences 111613 , Harbin, People's Republic of China; 2 Jiangsu Co-innovation Center for Prevention and Control of Important Animal Infectious Diseases and Zoonoses, Yangzhou, People's Republic of China; University of Freiburg, Freiburg, Germany

**Keywords:** rabies virus, receptor stability, GRP78

## Abstract

The rabies virus (RABV) is known to have four potential receptors, including the nicotinic acetylcholine receptor (nAChR), neural cell adhesion molecule (NCAM), low-affinity nerve growth factor receptor (NGFR), and metabolic glutamate receptor 2 (mGluR2). However, the regulatory mechanism that maintains the stability of these receptors on the cell membrane remains unclear. In this study, we found that 78 kDa glucose-regulated protein (GRP78) is required for RABV infection in cells. Knockdown of GRP78 decreased the internalization of RABV. A GRP78 monoclonal antibody effectively blocked RABV infection. Mechanistically, GRP78 interacts with RABV candidate receptors directly through the 19 to 50 amino acids at the N-terminal of GRP78, which is crucial for RABV infection. GRP78 can form complexes with viral receptors and viral envelope glycoproteins on the cell membrane. Silencing GRP78 led to a decrease in the expression of RABV candidate receptors on the cell membrane. Our findings suggest that GRP78 plays a critical role in stabilizing the cell-surface expression of these receptors by directly interacting with them, which in turn facilitates the internalization of RABV. These findings provide new insights into the complex process of RABV invasion into cells and may contribute to the development of therapeutics against RABV.

**IMPORTANCE:**

Rabies virus (RABV) is a neglected but fatal zoonotic virus. Research has shown that the RABV glycoprotein binds to cell receptors and, with host factors’ cooperation, invades cells to initiate its life cycle. Although several potential receptors and host factors for RABV have been identified, the process of virus invasion remains unclear. Here, we report that GRP78 has a significant inhibitory effect on RABV infection and plays a crucial role in its early stage, particularly during virus internalization. Furthermore, we found that GRP78 directly interacts with all candidate receptors of RABV, and this interaction is critical for virus internalization. Our study reveals how GRP78 mediates RABV invading cells and deepens our understanding of host-virus interaction mechanisms during RABV infection, providing a promising target for preventing and treating rabies.

## INTRODUCTION

Rabies is a fatal zoonotic disease induced by rabies virus (RABV) infection. Presently, there are no efficacious therapeutic drugs available for rabies. Once clinical symptoms emerge, the mortality rate is nearly 100%. Annually, rabies causes 59,000 deaths in more than 150 countries. However, due to inadequate reporting, this figure may be considerably underestimated. Rabies mainly occurs in economically underdeveloped countries where there is a dearth of infrastructure for timely reporting and post-exposure prophylaxis. Moreover, the extensive existence of livestock and wildlife hosts makes eradication highly improbable (1).

RABV is a negative-stranded RNA virus, with glycoprotein (G) being the only protein embedded on the envelope membrane of RABV. The virus binds to cells through its G protein interacting with receptors, thus initiating viral infection. It is known that RABV enters cells through receptor(s)-mediated clathrin-mediated endocytosis (CME) (2, 3). Four potential RABV receptors have been identified: nicotinic acetylcholine receptor (nAChR) (4), neural cell adhesion molecule (NCAM) (5), low-affinity nerve growth factor receptor (NGFR) (6), and metabolic glutamate receptor 2 (mGluR2) (7). In addition to the four potential receptors, other factors involved in RABV entry have also been identified, including transferrin receptor protein 1 (8, 9), AP-2-associated protein kinase 1 (10), integrin β1 (11), and heparan sulfate proteoglycans (12).

The initial stage of viral infection involves the binding of receptors on the cell membrane. However, the receptor is not firmly anchored to the cell membrane. Instead, receptors for some viruses shuttle between the plasma membrane and endosomes via the membrane trafficking machinery (13). Research has shed light on the role of E-cadherin and tumor-associated calcium signal transducer 2 (TACSTD2) in hepatitis C virus (HCV) infection. It has been found that this protein can modulate the localization of claudin-1 (CLDN1), which serves as a co-receptor for HCV on the cell surface (14–16)**,** ensuring that CLDN1 is stable on the cell surface. Similarly, modulation of neuropilin-1 has been shown to influence preS1 binding to the hepatitis B virus (HBV) receptor, sodium taurocholate cotransporting polypeptide (NTCP), which in turn regulates HBV infection (17). These studies demonstrate that the stable surface expression of the receptor is essential for efficient viral infection. However, the regulatory mechanism governing the stability of potential RABV receptors on the cell membrane remains to be elucidated.

GRP78 is a key endoplasmic reticulum chaperone that plays a central role in protein folding and endoplasmic reticulum quality control (18). Notably, GRP78 is implicated in regulating the infection and replication of multiple pathogens in host cells: Middle East respiratory syndrome coronavirus and bat coronavirus (HKU9) both mediate their attachment to host cells via GRP78 (19); GRP78 acts as a specific receptor for dengue virus serotype 2 during its infection of human hepatocellular carcinoma cells (HepG2) (20); it also plays an essential role in the invasion and replication of Japanese encephalitis virus in host cells (21); additionally, during coxsackievirus internalization, GRP78 synergizes with major histocompatibility complex class I molecules to act as a co-receptor for viral invasion (22). In this study, we demonstrate that GRP78 promotes RABV infection and plays a critical role during the viral internalization stage. It is noteworthy that GRP78 on the cell membrane blocks viral infection. Our research demonstrates that GRP78 facilitates viral infection by regulating the stability of RABV receptors on the cell membrane. This discovery deepens our understanding of the invasion process of RABV into cells and provides a promising target for the development of anti-RABV drugs.

## RESULTS

### GRP78 plays an important role in RABV infection

**Fig 1:**
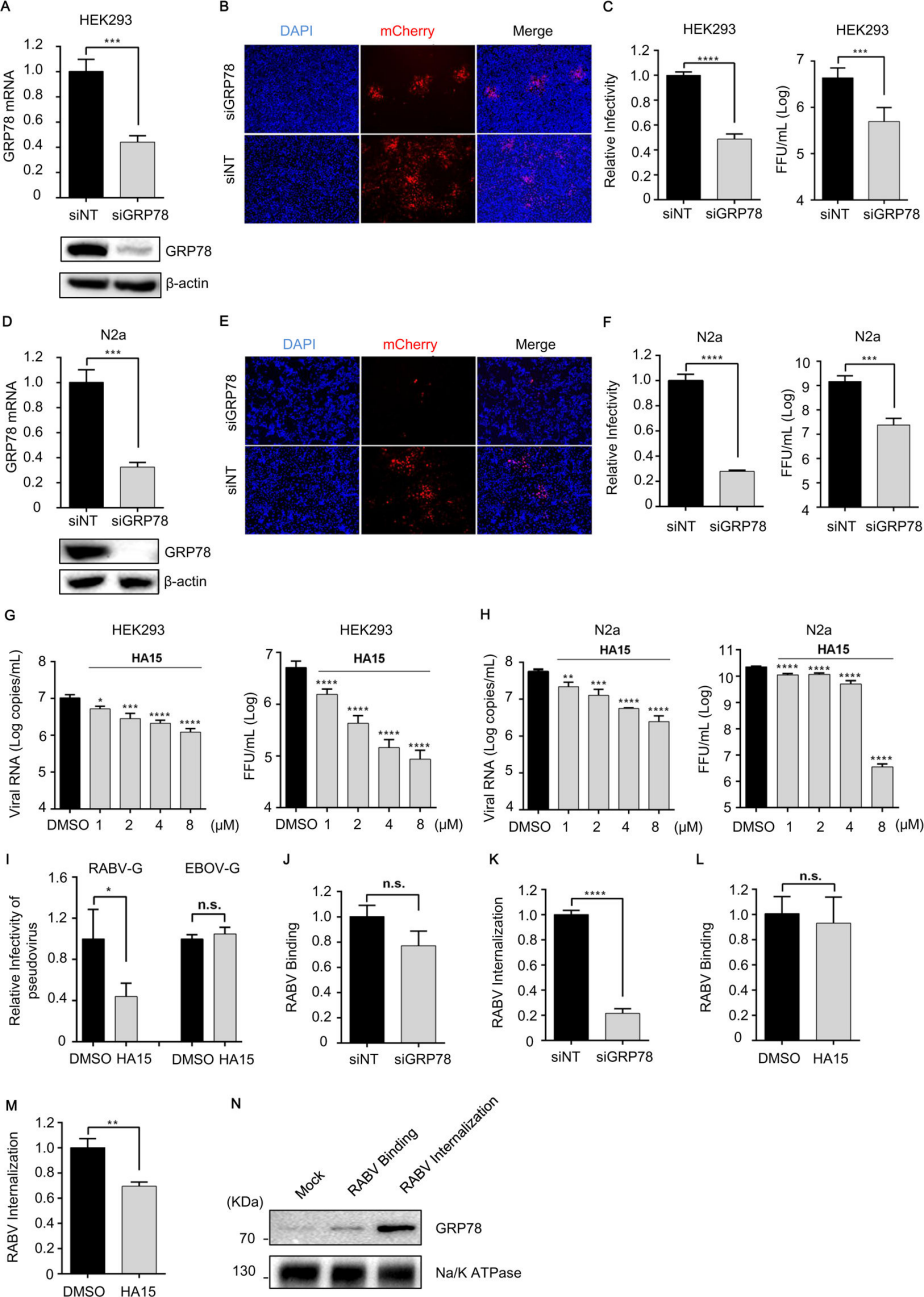
GRP78 involvement in RABV internalization. (**A and D**) The GRP78 mRNA in the GRP78 siRNA (siGRP78)-transfected HEK293 cells (A, upper panel) or N2a cells (D, upper panel) was measured by qPCR, and normalized to the NT siRNA (siNT)-transfected cells. GRP78 expression level in HEK293 cells (A, lower panel) or N2a cells (D, lower panel) was determined by Western blotting. β-Actin was used as a loading control. (**B and E**) The representative images of infected HEK293 cells (B) and N2a cells (E) are shown. (**C and F**) A high-content quantitative image-based analysis was taken at 48 h post-infection to measure the relative infection rate (normalized to siNT-treated cells) of ERA-mCherry in HEK-293 cells (C, left panel) and N2a cells (F, left panel) are shown. The cell supernatants were collected, and viral titers were quantified via endpoint dilution titration on BSR-T7/5 cells to determine the levels of ERA-mCherry in HEK293 (C, right panel) and N2a cells (F, right panel). (**G and H**) HEK293 cells or N2a cells were pretreated with indicated concentrations of HA15 or DMSO before infection with ERA-mCherry at an MOI of 0.01. Cell supernatant of HEK293 cells (G, left panel) or N2a cells (H, left panel) was harvested at 48 h post-infection for quantification of the viral RNA. The cell supernatants were collected, and viral titers were quantified via endpoint dilution titration on BSR-T7/5 cells to determine the levels of ERA-mCherry in HEK293 (G, right panel) and N2a cells (H, right panel). (**I**) HEK293 cells (left panel) or Vero-E6 cells (right panel) were pretreated with 5 μM HA15 prior to infection with HIV-based pseudovirions bearing the RABV glycoprotein (RABV-G) or EBOV glycoprotein (EBOV-G). Pseudovirus entry was quantified at 48 h post-infection, by measuring the luciferase signal. RABV binding (**J**) and internalization (**K**) assays were performed in GRP78-silenced N2a cells. Viral binding or internalization was quantified by normalization to the respective control cells. RABV binding (**L**) and internalization (**M**) assays were performed in HA15-treated N2a cells. Total RNA was extracted, and viral RNA was measured by qPCR. (**N**) N2a cells were bound or infected with RABV. The cell membrane was extracted and the content of GRP78 was detected by Western blotting. The Na/K ATPase was used as loading control. The two-tailed unpaired Student’s *t*-test was used for the statistical analysis in panels A, C, D, F, I, J, K, L, and M. All data represent the mean ± SD from three repeats. A one-way ANOVA was used for the statistical analysis in panels G and H. *, *P* < 0.05, **, P < 0.01, ***P < 0.001, ****, P < 0.0001; n.s., not significant.

Based on our previous investigations, a high-throughput screening of a siRNA library of the human genome was performed to identify host factors that exert an impact on RABV infection in HEK293 cells. It was found that silencing the expression of GRP78 can effectively inhibit RABV infection. To validate the effect of GRP78, siRNA targeting GRP78 (siGRP78) was transfected into different mammalian cell lines—human embryonic kidney cells (HEK293) and mouse neuroblastoma cells (N2a)—to knock down the expression of GRP78 in cells. Non-target siRNA (siNT)-transfected cells were used as negative controls. Twenty-four hours post-transfection, the expression level of GRP78 in siRNA-transfected cells was detected by Western blotting using specific antibodies against GRP78. It was observed that the expression of GRP78 protein in HEK293 cells (Fig. 1A, lower panel) and N2a cells (Fig. 1D, lower panel) transfected with siGRP78 significantly decreased, indicating successful silencing of GRP78 expression in these cell lines. Cells transfected with either siGRP78 or siNT were collected 24 h post-transfection, and total RNA was extracted for real-time quantitative polymerase chain reaction (qPCR). The results showed that mRNA levels for GRP78 were reduced by 56% in HEK293 cells (Fig. 1A, upper panel) and by 68% in N2a cells (Fig. 1D, upper panel) transfected with siGRP78, confirming the successful silencing of GRP78 protein expression in these two cell lines. Additionally, no significant difference in cell viability was observed between HEK293 cells (Fig. S1A) and N2a cells (Fig. S1B) transfected with siGRP78 compared to those transfected with siNT.

Subsequently, HEK293 cells and N2a cells were transfected with either siGRP78 or siNT and then infected with ERA-mCherry at a multiplicity of infection (MOI) of 0.1. The infection rate of RABV was determined by assessing the red fluorescence expressed in the cells using fluorescence imaging. It was observed that the cells transfected with siGRP78 exhibited less red fluorescence compared to those transfected with siNT (Fig. 1B and E), indicating a significant reduction in RABV infection upon silencing of GRP78 expression. Following fixation of the cells with paraformaldehyde and imaging using a high-content cell imaging system, statistical analysis revealed a 52% reduction in RABV infection rate in HEK293 cells (Fig. 1C, left panel) and a 72% reduction in N2a cells (Fig. 1F, left panel) after knockdown of GRP78. Meanwhile, cell supernatants were harvested and assayed for viral titer via titration on BSR-T7/5 cells. The results demonstrated that the level of ERA-mCherry infection was significantly reduced in HEK293 and N2a cells transfected with GRP78 siRNA, compared with NT siRNA-transfected counterparts (Fig. 1C, right panel; Fig. 1F, right panel, respectively). The above results demonstrate that silencing GRP78 has a substantial inhibitory effect on RABV infection across various cell types.

Previous research has identified HA15 as a chemical molecule that specifically targets GRP78 (23). To further investigate the impact of GRP78 on RABV infection, HEK293 and N2a cells were pretreated with different concentrations of HA15 or dimethyl sulfoxide (DMSO) for 1 h at 37°C prior to being infected with ERA-mCherry at an MOI of 0.01. The cell supernatant was collected 48 h post-infection, and the RABV copies were detected by qPCR. As shown in Fig. 1G and H, HA15 significantly inhibited ERA-mCherry infection in HEK293 cells (Fig. 1G, left panel) and N2a cells (Fig. 1H, left panel) in a dose-dependent manner. Collected cell supernatants were subjected to viral titer quantification using BSR-T7/5 cells. The results demonstrated that ERA-mCherry infection levels were significantly reduced in HA15-treated HEK293 and N2a cells relative to DMSO-treated control cells (Fig. 1G, right panel; Fig. 1H, right panel, respectively). At the same time, the effects of different concentrations of HA15 on cell viability in HEK293 cells and N2a cells were tested. All detected concentrations of HA15 had no inhibitory effect on the viability of HEK293 cells and N2a cells (Fig. S1C and D).

### GRP78 promotes the internalization of RABV

The envelope G protein of RABV is the only protein present on the virus surface, mediating the invasion of the virus. To determine whether GRP78 influences RABV invasion, we carried out infection experiments using a pseudovirus with HIV as the backbone for packaging the RABV G protein. Pseudoviruses bearing the Ebola virus glycoprotein (EBOV-G) were used as a negative control. HEK293 or Vero-E6 cells pretreated with 5 µM HA15 (a specific inhibitor of GRP78) or DMSO were infected with the two pseudoviruses, respectively. At 48 h post-infection, cell lysates were collected, and firefly luciferase activity was measured using a commercial assay kit from Promega. We found that HA15 treatment significantly reduced the infection efficiency of RABV-G pseudoviruses but had no effect on EBOV-G pseudoviruses (Fig. 1I). These results indicate that GRP78 specifically regulates the early stage of RABV infection.

To determine at which stage (binding or internalization) GRP78 inhibits RABV invasion, the following experiments were conducted. For identifying the effect of GRP78 on virus binding, N2a cells were transfected with either siGRP78 or siNT and then infected with ERA-mCherry at a MOI of 1. The cell culture dishes were placed on ice for 1 h to facilitate virus attachment and binding to the cells. Subsequently, the unbound virus was removed using PBS, and total RNA of the cells was extracted. To assess the impact of GRP78 on virus internalization, the cells were incubated at 37°C for an additional hour after initial virus binding on ice to allow RABV internalization. Then, the unbound virus was washed out with PBS and trypsin, followed by extraction of total RNA from the cells. The presence of the RABV N gene in the cells was detected using qPCR. It was observed that silencing GRP78 did not affect the binding of RABV to the cells (Fig. 1J) but significantly reduced virus internalization into the cells (Fig. 1K). Furthermore, N2a cells treated with HA15 (5 μM) showed no influence on RABV binding to the cells (Fig. 1L), but exhibited a significant reduction in internalized virus particles within the cells (Fig. 1M).

Subsequently, we investigated whether there were changes in GRP78 levels on the cell membrane during the early stages of RABV infection. N2a cells were incubated with RABV (MOI = 5) on ice for 1 h to allow virus adsorption and binding, followed by infection at 37°C for another hour. After three washes with PBS to remove the cell surface-bound viruses, cell membrane extraction was performed and the GRP78 content on the membrane was assessed via Western blotting. It was found that endogenous GRP78 levels significantly increased on infected cell membranes (Fig. 1N).

### Blocking of GRP78 with an antibody inhibits RABV infection

**Fig 2:**
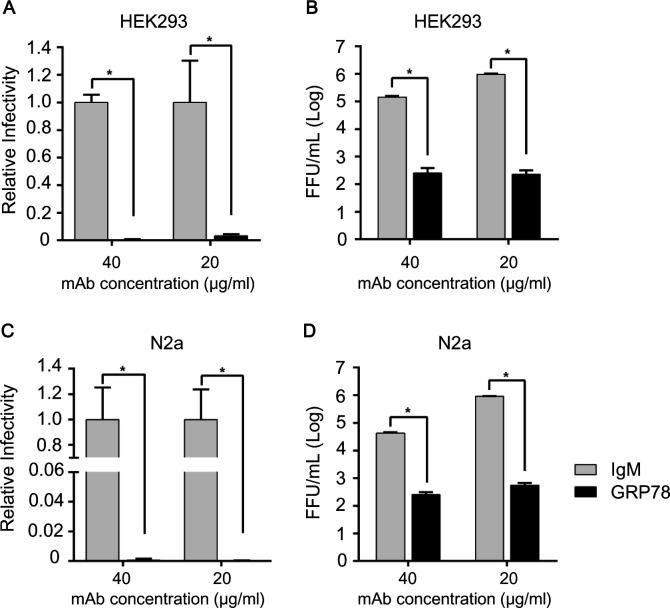
The cell-surface GRP78 is required for RABV infection. (**A and C**) The monoclonal antibody (mAb) against GRP78 blocked ERA-mCherry infection. The percentages of infected HEK293 cells (A) and N2a cells (C) are shown. (**B and D**) The mAb against GRP78 decreased the replication of ERA-mCherry in HEK293 cells (B) and N2a cells (D). Virus titers in the cell culture supernatant were determined as focus-forming units in BSR-T7/5 cells. The isotype IgM at the same concentration was used as controls for the mAb. Values represent the mean ± SD. A two-tailed unpaired Student’s *t*-test was used for the statistical analysis. *, *P* < 0.05*.*

GRP78 can localize both in the cytoplasm and on the outermost layer of the cell (24, 25). The aforementioned experiments have demonstrated that GRP78 is involved in the internalization of RABV, indicating that the GRP78 expressed on the outermost layer of the cell is important for RABV infection. To determine whether extracellular GRP78 has an effect on RABV internalization, we conducted antibody blocking experiments. The antibody blocking assay was conducted in HEK293 cells and N2a cells, respectively. After incubating with different concentrations of GRP78 monoclonal antibodies or the antibody isotype control, ERA-mCherry (MOI = 0.1) was added and co-incubated with the cells on ice for 1 h. Subsequently, unbound virus was washed off with PBS, and fresh culture medium was added to the cells. After 48 h, the cell culture supernatant was collected for virus titration, and the cells were fixed with paraformaldehyde and photographed using a high-content cell imaging system. Based on the analysis of fluorescence intensity in cells by Columbus software (PerkinElmer, Waltham, MA, USA), it was observed that the relative infection rate decreased almost 100% after treatment with both concentrations of GRP78 antibody in HEK293 cells (Fig. 2A). Similarly, in N2a cells treated with GRP78 antibody, GRP78 mAb achieved almost 100% inhibition for both concentrations tested (Fig. 2C). The virus titers significantly decreased compared to the isotype control at both antibody concentrations in HEK293 cells (Fig. 2B) and N2a cells (Fig. 2D). The cell viability of HEK293 cells treated with GRP78 antibody or its isotype control showed no significant difference (Fig. S2A), while the viability of N2a cells was reduced by 20% (Fig. S2B). Since antibody molecules cannot enter unpermeabilized cells, blocking the GRP78 protein on the cell membrane can reduce RABV infection. The results suggest that reducing the GRP78 protein on the cell membrane can inhibit RABV infection, especially in the early stages.

### GRP78 indirectly interacts with the RABV G protein

**Fig 3:**
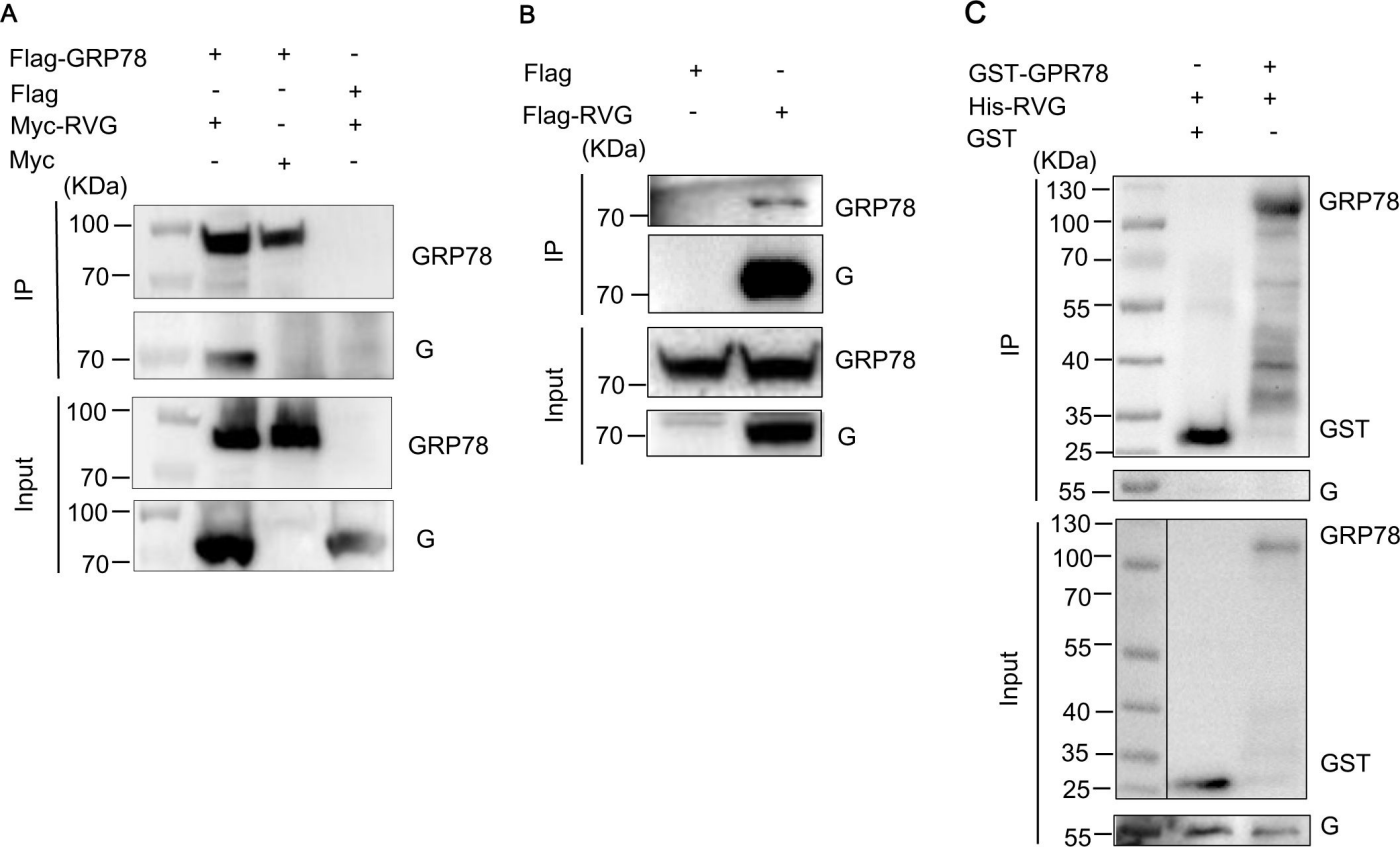
GRP78 interacts with RABV G protein. (**A**) HEK293 cells were co-transfected with Flag-GRP78 and Myc-RVG plasmids. Cell lysates were subjected to Co-IP using anti-Flag agarose beads. Immunoprecipitants and whole-cell lysates (Input) were immunoblotted with anti-Flag and anti-Myc antibodies, respectively. (**B**) Co-IP assay was performed to examine the interaction between Flag-RVG and endogenous GRP78 in HEK293 cells. (**C**) Purified GST-GRP78 protein was incubated with purified His-RVG protein, followed by a pull-down assay using anti-GST beads. GST protein alone was used as a negative control.

The envelope G protein of RABV is the only protein present on the virus surface, mediating the viral entry process. To test whether GRP78 mediates RABV entry through directly interacting with the G protein, we carried out co-immunoprecipitation (Co-IP) experiments to verify if there exists an interaction between GRP78 and the G protein. For the Co-IP assay, HEK293 cells were transfected with plasmids encoding Flag-tagged GRP78 (Flag-GRP78) and Myc-tagged RABV G (Myc-RVG). The results demonstrated that GRP78 interacts with RABV G (Fig. 3A).

We transfected Flag-tagged RABV G (Flag-RVG) plasmids into HEK293 cells and detected its interaction with endogenous GRP78 in the cells through immunoprecipitation (IP). The results indicate that the RABV G protein interacts with endogenous GRP78 (Fig. 3B).

Furthermore, pull-down assays were carried out to determine if RABV G directly interacts with GRP78. GST-tagged GRP78 (GST-GRP78) and His-tagged RABV G ectodomain (His-RVG) were purified separately and then pooled for pull-down assays. The results showed that there is no direct interaction between the ectodomain of RABV G and GRP78 (Fig. 3C), suggesting that GRP78 interacts with RABV G indirectly.

### GRP78 interacts with RABV receptors

**Fig 4:**
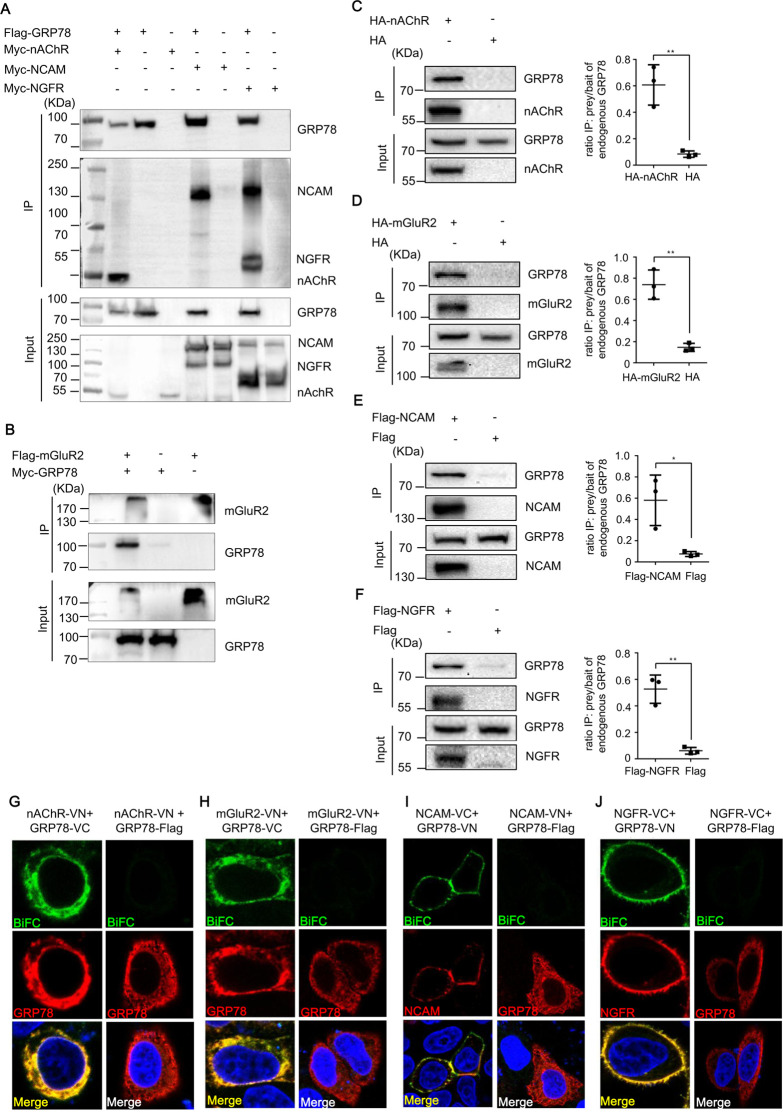
GRP78 interacts with RABV receptors. (**A and B**) HEK293 cells were co-transfected with the following plasmid combinations: (A) Flag-GRP78 plus Myc-nAChR, Myc-NCAM, or Myc-NGFR; (B) Flag-mGluR2 plus Myc-GRP78. After transfection, cell lysates were subjected to IP using anti-Flag agarose beads. Representative Western blotting results of whole-cell lysates (Input) and IP eluates are shown. (**C–F**) HEK293 cells were transfected with HA-nAChR (C), HA-mGluR2 (D), Flag-NCAM (E), or Flag-NGFR (F) plasmids. Cell lysates were subjected to IP using anti-HA or anti-Flag beads, respectively. Immunoprecipitants and whole-cell lysates (Input) were analyzed by immunoblotting with anti-HA, anti-Flag, and anti-GRP78 antibodies. Representative images are presented. Band intensities of endogenous GRP78 in bait and prey samples from three independent Western blotting experiments were quantified using ImageJ, and statistical analysis was performed with GraphPad Prism. Statistical significance was determined using a two-tailed unpaired Student’s *t*-test. *, P < 0.05, **, P < 0.01*.* (**G–J**) Representative confocal images showing the subcellular localization of GRP78 and RABV receptors. GRP78-VC (G and H, left panels), NCAM-VC (I, left panel), NGFR-VC (J, left panel), and GRP78-Flag (G to J, right panels) were immunostained with a rabbit anti-Flag primary antibody, followed by incubation with a TRITC-conjugated goat anti-rabbit secondary antibody. The co-localization of red fluorescent signals (from TRITC staining) and green fluorescent signals (from BiFC) was visualized using confocal laser scanning microscopy.

The viral receptors are responsible for virus entry. As GRP78 does not directly interact with the RABV G protein, we guessed whether GRP78 regulated RABV internalization via interacting with RABV receptors. Until now, four potential RABV receptors have been identified: nAChR, NCAM, NGFR, and mGluR2. We first investigated whether there exists an interaction between GRP78 and these receptors of RABV by Co-IP assays. HEK293 cells were transfected with plasmids of Flag-tagged GRP78 (Flag-GRP78) and the known RABV receptors NCAM (Myc-NCAM), NGFR (Myc-NGFR), nAChR (Myc-nAChR), as well as Myc-tagged GRP78 (Myc-GRP78) and Flag-tagged mGluR2 (Flag-mGluR2). The results demonstrated interactions between GRP78 and all known RABV receptors thus far (Fig. 4A and B).

Furthermore, HA-mGluR2, Flag-NCAM, Flag-NGFR, and HA-nAchR plasmids were transfected into HEK293 cells to examine their interaction with endogenous GRP78 by IP. The results in Fig. 4C through F demonstrate that nAChR, nNCAM, NGFR, and mGluR2 all interact with endogenous GRP78.

To explore the interaction between GRP78 and receptors of RABV in a cellular context, a bimolecular fluorescence complementation (BiFC) assay was conducted. The green fluorescent protein Venus was split into two complementary fragments, namely the N-terminal residues of Venus (VN) and the C-terminal residues of Venus (VC). These fragments were fused with an interacting protein pair. When the protein-protein interaction takes place within the same cell, the two fragments of the Venus protein fuse due to proximity and emit a fluorescent signal. Moreover, this fluorescent signal indicates the location of the protein-protein interaction (26). HeLa cells were co-transfected with plasmids encoding GRP78-VC and nAChR-VN. After 24 h, the cells were fixed with paraformaldehyde. Subsequently, an indirect immunofluorescence assay (IFA) was performed. Rabbit anti-Flag antibody was used as the primary antibody to label GRP78-VC, and TRITC-labeled anti-rabbit IgG antibody was employed as the secondary antibody for staining. By observing the fluorescence using a confocal microscope, it was found that the green fluorescence emitted by the interaction between GRP78-VC and nAChR-VN is predominantly localized in the cytoplasm. Moreover, GRP78 (red) is also mainly expressed in the cytoplasm (Fig. 4G, left panel). Plasmids encoding mGluR2-VN and GRP78-VC were transfected by employing the same approach. Subsequently, IFA was conducted to label GRP78-VC. We observed that mGluR2 and GRP78 interact within the cytoplasm, as shown in Fig. 4H, left panel. The interaction between NCAM-VC and GRP78-VN is localized on the cell membrane, as depicted in Fig. 4I, left panel. Likewise, the interaction between NGFR-VC and GRP78-VN is also situated on the cell membrane, as indicated in Fig. 4J, left panel. As negative controls, co-transfection of GRP78-Flag with nAChR-VN, mGluR2-VN, NCAM-VN, or NGFR-VC did not yield detectable green fluorescence (Fig. 4G through J, right panels).

Based on the aforementioned experimental results, we can draw the conclusion that GRP78 directly interacts with the candidate receptors of RABV.

### The 19 to 50 amino acids (aa) at the N-terminal of GRP78 are the key regions for interacting with RABV receptors

**Fig 5:**
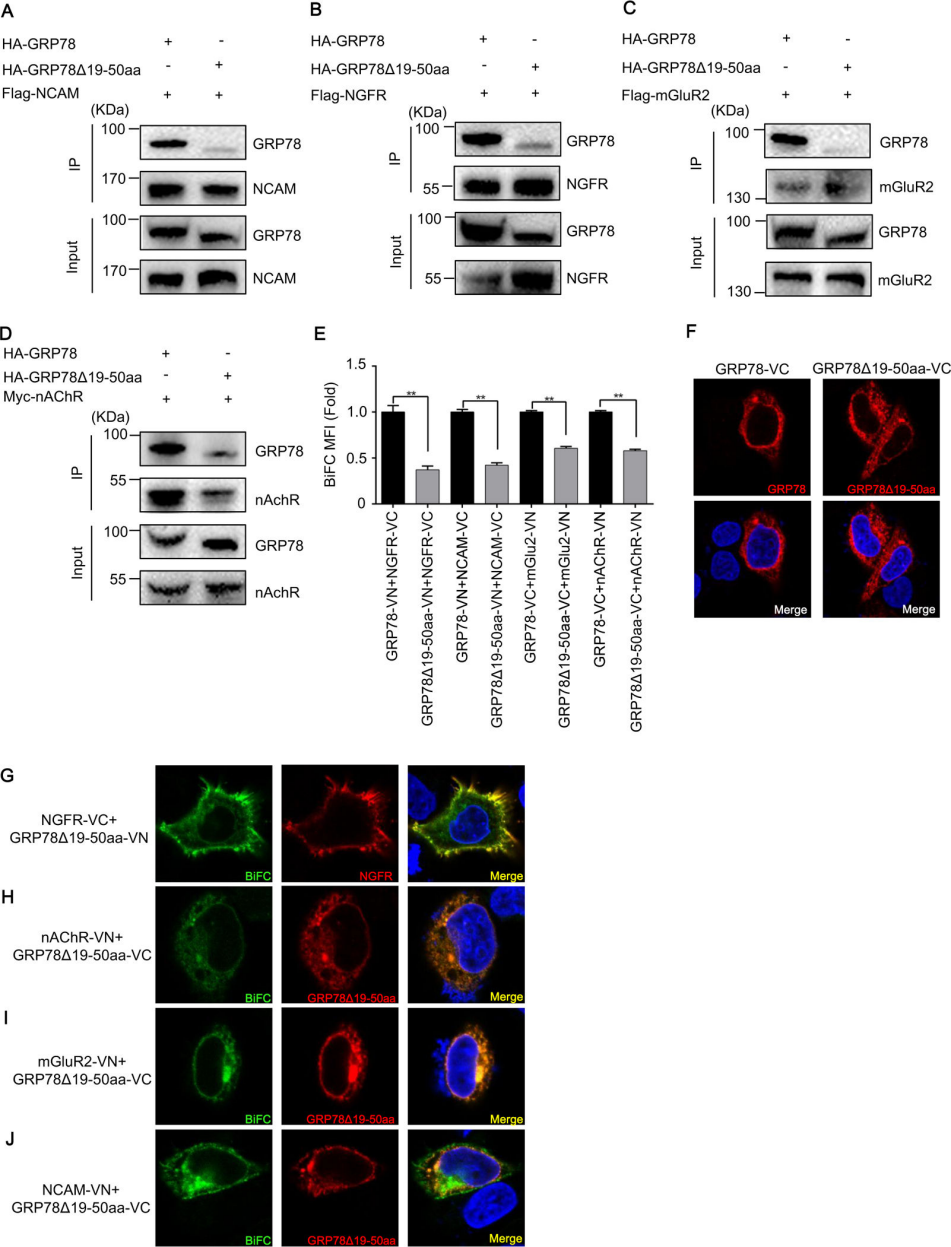
The N-terminal 19–50 aa of GRP78 is the key region that interacts with RABV receptors. (**A–D**) The Co-IP assay was carried out to explore the interaction of HA-GRP78 or HA-GRP78Δ19–50aa, with Myc-NGFR, Myc-NCAM, Myc-nAChR, and Flag-mGluR2. (**E**) GRP78-VN, GRP78Δ19–50aa-VN, GRP78-VC, and GRP78Δ19–50aa-VC were individually paired with their complementary BiFC partners, including NGFR-VC, NCAM-VC, nAChR-VN, and mGluR2-VN, respectively. Fluorescent signals were detected by flow cytometry. The BiFC mean fluorescence intensity (MFI) was normalized to the signal from WT GRP78-transfected cells. All data are presented as the mean ± SD from two independent experiments. A one-way ANOVA was used for the statistical analysis. **, *P* < 0.01. (**F**) Representative immunofluorescence images of GRP78 and GRP78Δ19–50aa expressed in cells. GRP78-VC (left panels) and GRP78Δ19–50aa-VC (right panel) were immunostained with a rabbit anti-Flag antibody, followed by a TRITC-conjugated goat anti-rabbit secondary antibody. Red fluorescent signals were visualized using confocal microscopy. (**G–J**) Representative confocal microscopy images showing the subcellular localization of GRP78Δ19–50aa and RABV receptors. NGFR-VC (panel G) and GRP78Δ19–50aa-VC (panels H to J) were immunostained with a rabbit anti-Flag primary antibody, followed by a TRITC-conjugated goat anti-rabbit secondary antibody. Co-localization of the red (immunofluorescence) and green (BiFC) fluorescent signals was visualized using confocal microscopy.

We next sought to identify the key regions on GRP78 that influence its interaction with RABV receptors. In the antibody blocking assays performed in the present study (Fig. 2), we found that antibodies targeting aa 19–50 within the N-terminus of GRP78 could inhibit RABV infection. To further define the key domains within GRP78, the amino acids at positions 19 to 50 of the N-terminus were deleted, resulting in truncated GRP78 (GRP78Δ19–50aa). Subsequently, plasmid constructs fused with genes for GRP78Δ19–50aa and Flag or HA tags were generated. A Co-IP assay was performed to investigate whether the interaction between GRP78 and RABV receptors depends on this domain of GRP78. After transfection of the truncated or wild-type (WT) plasmids into HEK293 cells, the input proteins in the whole cell lysate were expressed at similar levels. Compared with WT GRP78, the GRP78Δ19–50aa bound to RABV receptors at a lower level (Fig. 5A through D).

When WT GRP78-VN or GRP78-VC was paired with the complementary NGFR-VC, NCAM-VC, nAChR-VN, and mGluR2-VN BiFC partners, strong fluorescent signals were detected by flow cytometry. However, when GRP78Δ19–50aa-VN and GRP78Δ19–50aa-VC were paired with the complementary NGFR-VC, NCAM-VC, nAChR-VN, and mGluR2-VN BiFC partners, the fluorescent signals detected were significantly weakened compared to WT GRP78 (Fig. 5E). This proves that the amino acids ranging from 19 to 50 at the N-terminal of GRP78 are key regions that influence its interaction with RABV receptors.

To determine whether deletion of 19–50 aa in the GRP78 protein impacts its subcellular localization, we transiently transfected HEK293 cells with GRP78-VC (WT) and GRP78Δ19–50aa-VC plasmids, respectively. Subcellular localization of GRP78 was then assessed via IFA. As depicted in Fig. 5F, GRP78Δ19–50aa displayed an identical subcellular localization pattern to WT GRP78, demonstrating that removal of 19–50 aa does not alter the subcellular localization of GRP78.

To characterize the subcellular localization of BiFC complexes, we co-transfected HeLa cells with either GRP78Δ19–50aa-VN or GRP78Δ19–50aa-VC, together with their respective BiFC partners (NGFR-VC, NCAM-VC, nAChR-VN, and mGluR2-VN). The localization patterns of the resultant complexes were subsequently visualized by confocal fluorescence microscopy. As illustrated in Fig. 5G through J, the colocalization of GRP78Δ19–50aa with NGFR, NCAM, nAChR, and mGluR2 was identical to that of WT GRP78, demonstrating that deletion of amino acids 19–50 does not elicit any detectable alteration in the colocalization profile of GRP78 with its interaction partners.

We demonstrated that amino acids 19–50 within the N-terminal domain of GRP78 constitute the key region mediating its interaction with the RABV receptor.

### GRP78 stabilizes the cell surface expression of RABV receptors

**Fig 6:**
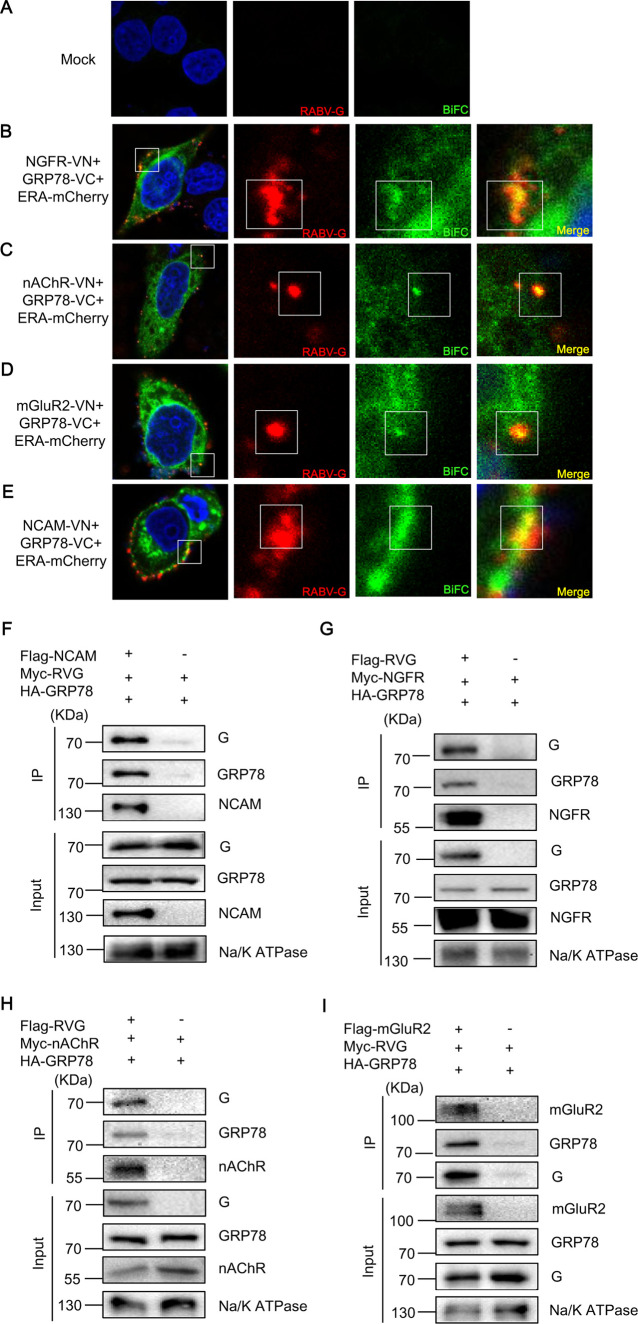
GRP78 interacts with RABV G protein and RABV receptors on the cell membrane. (**A–E**) Representative confocal microscopy images showing the subcellular localization of GRP78, RABV G protein, and RABV receptors. HeLa cells were co-transfected with plasmids encoding distinct BiFC partner pairs: nAChR-VN/GRP78-VC, mGluR2-VN/GRP78-VC, NCAM-VN/GRP78-VC, and NGFR-VN/GRP78-VC. At 24 h post-transfection, cells were incubated with ERA-mCherry (MOI = 5) at 4°C for 1 h to facilitate viral attachment, followed by a 10 min incubation at 37°C to trigger viral internalization. Subsequently, RABV G protein was immunostained with a mouse anti-RABV G monoclonal antibody, followed by a TRITC-conjugated goat anti-mouse secondary antibody. Co-localization of the red (RABV) and green (BiFC) fluorescent signals was visualized using confocal microscopy. (**F–I**) HEK293 cells were transfected with the plasmids of GRP78, RABV G, and RABV receptors. The cell membrane was extracted at 48 h post-transfection, and the membrane components were bound to anti-Flag beads. A Co-IP assay was performed to detect the interaction between them.

We further investigated the impact of GRP78 on RABV receptors localized to the cell membrane. To this end, we first performed BiFC assays to verify whether GRP78, RABV receptors, and the viral G protein interact with one another on the cell surface during RABV internalization. HeLa cells were co-transfected with plasmids encoding distinct BiFC partner pairs, namely nAChR-VN/GRP78-VC, mGluR2-VN/GRP78-VC, NCAM-VN/GRP78-VC, and NGFR-VN/GRP78-VC. At 24 h post-transfection, cells were incubated with ERA-mCherry (MOI = 5) at 4°C for 1 h to facilitate viral attachment, followed by a 10 min incubation at 37°C to trigger viral internalization. Subsequently, RABV G protein was detected via IFA, and the colocalization among these three components was examined using confocal fluorescence microscopy. Confocal imaging confirmed the colocalization of green-fluorescent BiFC complexes (nAChR-VN/GRP78-VC, mGluR2-VN/GRP78-VC, NCAM-VN/GRP78-VC, and NGFR-VN/GRP78-VC) with red-fluorescent RABV G protein on the cell membrane (Fig. 6A through E). Collectively, these results demonstrate that GRP78 interacts with both the RABV G protein and its cognate receptors to form a ternary complex on the cell membrane during viral internalization.

To confirm the formation of a complex comprising GRP78, the RABV G protein, and the RABV receptors on the cell membrane, we transfected plasmids encoding these proteins into HEK293 cells. Subsequently, at 48 h post-transfection, we isolated and extracted the cell membrane. The extracted components were then bound to Flag beads for a Co-IP assay, which confirmed interactions between GRP78, the RABV G protein, and the RABV receptors on the cell membrane (Fig. 6F through I).

**Fig 7:**
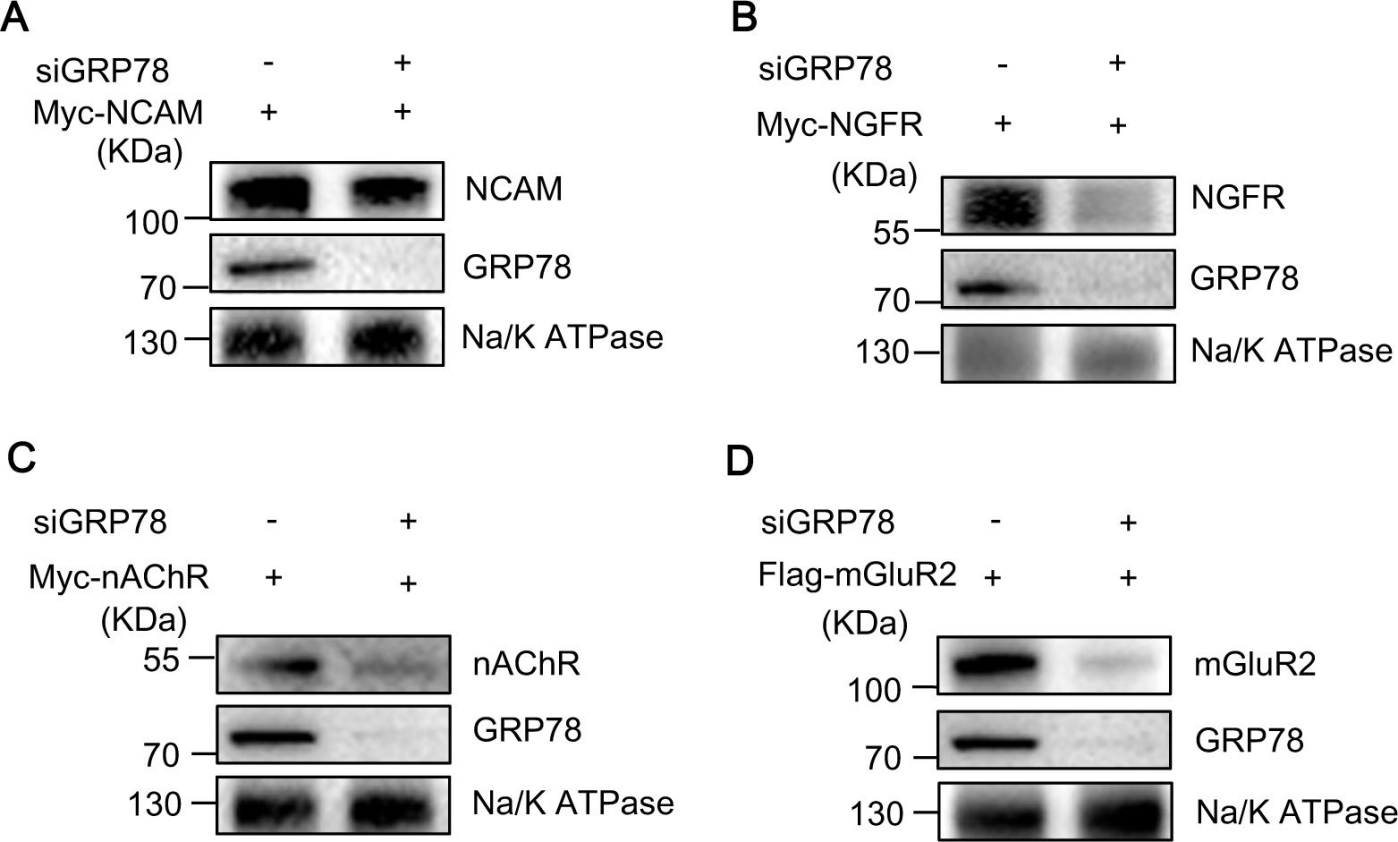
GRP78 stabilizes the cell-surface expression of RABV receptors. (**A–D**) N2a cells were transfected with siGRP78. At 24 h post-transfection, the cells were transfected with plasmids of RABV receptors. After another 24 h, cells were collected, and the cell membrane was extracted. The expression of GRP78 and RABV receptors on the cell membrane was detected by Western blotting. The Na/K ATPase was used as a loading control.

We next performed the expression of RABV receptors in control or GRP78-silenced N2a cells by Western blotting assays. N2a cells were transfected with siGRP78 or siNT. Subsequently, 24 h post-transfection, the plasmids encoding RABV receptors were transfected. Another 24 h later, the cells were harvested, and the cell membrane was extracted. The expression of GRP78 and RABV receptors on the cell membrane was detected by Western blotting. We observed a significant reduction in the expression of RABV receptors on the cell membrane upon knockdown of GRP78 (Fig. 7A through D), indicating that GRP78 facilitates RABV infection by stabilizing the expression of RABV receptors on the cell membrane.

## DISCUSSION

In this study, we first confirmed the crucial role of GRP78 in RABV infection and identified its regulatory effect on the viral internalization process. Specifically, we revealed the significance of cell membrane-localized GRP78 for RABV infection. Subsequently, we determined that the effect of GRP78 on RABV infection is not mediated by direct interaction with the viral envelope glycoprotein, but rather by direct interaction with currently known potential RABV receptors. Finally, we demonstrated that GRP78 can stabilize the membrane expression of RABV receptors, thereby facilitating viral infection.

While GRP78 is predominantly localized to the endoplasmic reticulum, it can also be detected on the cell surface (18, 25). The surface expression level of GRP78 is markedly upregulated under stress-inducing conditions, including viral infection. A growing body of evidence indicates that membrane-bound GRP78 plays a pivotal role in mediating viral entry. Specifically, membrane-bound GRP78 interacts with the envelope proteins of enveloped RNA viruses, thereby facilitating viral binding and internalization (19–22, 27–29). In the present study, we observed a notable elevation in the level of membrane-localized GRP78 during RABV internalization (Fig. 1N). Together with the results from GRP78 antibody blocking assays (Fig. 2A through D), these findings collectively corroborate the indispensable role of membrane-bound GRP78 in RABV invasion. We further demonstrated that GRP78 interacts directly with RABV receptors and verified that RABV receptor expression is significantly diminished in GRP78-silenced cell membranes. However, the underlying regulatory mechanism remains elusive: it remains unclear whether this reduction in RABV receptor expression stems from the depletion of membrane-localized GRP78 or from the regulatory effects exerted by GRP78 via its chaperone function. The detailed regulatory mechanisms involved herein await further exploration.

Previous studies have demonstrated that GRP78 modulates viral infection via direct interaction with viral glycoproteins, with its N-terminal amino acid residues 1–100 being implicated in multiple viral infection processes (21, 27). By comparison, our findings reveal that GRP78 mediates RABV infection through direct interaction with RABV receptors. Our study further identifies that amino acid residues 19–50 of GRP78 serve as the critical region responsible for binding to these receptors. Collectively, these results indicate that GRP78 employs distinct mechanisms to facilitate the entry of different viruses.

Although RABV solely invades cells through CME, it employs multiple potential cell receptors and host molecules to collaborate in this process. mGluR2 is currently the only receptor proven to function *in vitro* and *in vivo*. However, studies have shown that the mortality rate of mGluR2 knockout mice challenged with RABV (street virus GX/09) was approximately 42% (8), indicating that there are other host factors besides mGluR2 that mediate RABV infection *in vivo*. Blocking the early stages of RABV infection presents an attractive drug development strategy. Currently, there is a lack of therapeutic drugs for RABV exposure, and the only post-exposure prophylaxis available is through emergency injection of immunoglobulin. In this study, it was discovered that GRP78 interacts with four candidate receptors of RABV and has a regulatory effect on their expression. This provides a new direction for anti-RABV strategies by decreasing the expression of GRP78 to reduce the expression of all RABV receptors and inhibit viral infection. In this study, we also demonstrated that the specific inhibitor of GRP78, HA15, and the monoclonal antibody against GRP78 can inhibit RABV invasion. Notably, the peptide segment at the N-terminal of GRP78, spanning from amino acid residues 19 to 50, is crucial for the interaction between candidate receptors and GRP78. Given its significance in various viruses’ early life cycles, targeting GRP78 with safe and effective inhibitors holds promise for preventing and treating important zoonotic diseases.

## MATERIALS AND METHODS

### Cell and virus

HEK293 cells (ATCC CRL-1573), N2a cells (ATCC, CCL-131), HeLa cells (ATCC CCL-2), and Vero-E6 cells (ATCC, CRL-1586) were cultured in Dulbecco’s modified Eagle’s medium (DMEM) supplemented with 10% fetal bovine serum (FBS), L-glutamine, and penicillin-streptomycin. BSR-T7/5 (RRID: CVCL_RW96) cells, a BHK-derived cell line stably expressing T7 RNA polymerase (30), were obtained from the National Infrastructure of Cell Resources (Beijing, China) and maintained in our laboratory (31). The cells were cultured in DMEM supplemented with 5% FBS, L-glutamine, and 1% penicillin-streptomycin.

The Evelyn-Rokitnicki-Abelseth (ERA) strain of RABV, a vaccine strain derived from the original SAD isolate following serial passage and propagation in various cell lines (32), was obtained from the China Veterinary Culture Collection and has been maintained in our laboratory (33). ERA-mCherry is a recombinant ERA strain in which the mCherry gene is inserted between the ERA M and G genes as an additional transcription unit. This recombinant virus was constructed and preserved in our laboratory (10).

### RNA interference (RNAi)

The RNAi assay was conducted as previously described. Briefly, siRNA targeting the human GRP78 (10 pmol/well in 24-well plates; Ambion, s6981), mouse GRP78 siRNA (5 pmol/well in 24-well plates; Ambion, s67085), or non-targeting (NT) siRNA (Ambion, 4390843), was mixed with Opti-MEM medium containing 0.3 μL/pmol siRNA of Lipofectamine RNAiMAX transfection reagent in a volume of 160 μL per well in 24-well plates. After a 30 min incubation at room temperature, HEK293 cells or N2a cells were seeded into siRNA-coated 24-well plates at a density of 5 × 10^4^ cells/well in a volume of 500 μL per well. At 24 h post-siRNA transfection, total RNA was extracted from the cells, and the GRP78 mRNA level was determined by qPCR. The infected cells were then exposed to ERA-mCherry at 24 h post-transfection, and the infection rate was assessed at 48 h post-infection using the Perkin-Elmer Operetta high-content system.

### qPCR

To detect the GRP78 mRNA level and viral RNA level in cells, total RNA from cells was isolated using TRIzol reagent (Thermo Fisher). Total RNA was used for reverse transcription (Vazyme). Relative mRNA expression was analyzed by using SYBR Green qPCR Master Mix (Vazyme, Q711) with the indicated GRP78 and RABV N gene-specific primers. The 2^-ΔΔCt^ method was used to calculate the relative gene expression level, with GAPDH as the internal control. Experiments were done in three biological replicates. The qPCR primers used were as follows: GRP78, forward (5′-AACCATCCCGTGGCATAAA-3′) and reverse (5′-GGACATACATCAAGCAGTACCA-3′); RABV N gene, forward (5′-ACTAGGCTTGAGTGGGAAATC-3′) and reverse (5′-GGAGCACATGCAGCAATAAC-3′); GAPDH (human), forward (5′-CCTCCTGTTCGACAGTCAGC-3′) and reverse (5′-CGCCCAATACGACCAAATC-3′); GAPDH (mouse), forward (5′-CCTTCATTGACCTCAACTACATGG-3′) and reverse (5′-CTCGCTCCTGGAAGATGGTG-3′).

### Western blotting

Western blotting analysis was performed by diluting clarified cell lysate in denaturing SDS gel loading buffer and boiling for 10 min. The denatured samples were then loaded onto a 4 to 20% gel (GenScript, M00657) for SDS-PAGE and separated by electrophoresis. Proteins were transferred to a polyvinylidene difluoride (PVDF) membrane (Merck-Millipore, ISEQ00010). The PVDF membrane was blocked with 5% skim milk and incubated with primary antibodies against Flag (GenScript, A00170), Myc (GenScript, A00172), or HA (Sigma, H3663). After washing the PVDF membrane three times with PBST, it was incubated with horseradish peroxidase-conjugated goat anti-rabbit antibody (ZSBio, ZB-2301). Target protein bands were detected using enhanced chemiluminescence reagent (Merck Millipore, WBLUR0500).

### Inhibitor assay

For the inhibitor assay, N2a cells were preincubated with either HA15 (Selleck) at the indicated concentrations or DMSO for 1 h at 37°C. Subsequently, the cells were infected with ERA-mCherry virus at an MOI of 0.1 for 1 h at 37°C. After washing the cells, medium containing the specified inhibitor was added, and virus levels in the supernatant were measured by qPCR at 48 h post-infection.

### Pseudovirus packing

We used pNL4-3.Luc.R-E- to package the pseudovirus. The pNL4-3.Luc.R-E- is a plasmid based on the HIV-1 proviral clone pNL4-3. HEK293 cells were transfected with a mixture of plasmids encoding pNL4-3.Luc.R-E- and RABV glycoproteins or EBOV glycoproteins at a ratio of 1:1. Supernatants were harvested 48 h post-transfection, aliquoted, and frozen at −80°C. Subsequently, HEK293 cells or Vero-E6 cells were pretreated with either 5 µM HA15 or DMSO and then infected with pseudovirus for 2 h. At 48 h post-infection, cell lysates were collected, and the activity of firefly luciferase (Promega) was measured.

### Antibody blocking

In antibody blocking experiments on HEK293 cells and N2a cells, a mouse IgM antibody to GRP78 A-10 (Santa Cruz Biotechnology, sc-376768) was used. Mouse IgM (Santa Cruz Biotechnology, sc-53347) served as an isotype control. Cells on 96-well carrier plates were incubated with medium containing different concentrations of antibodies and isotype antibody (40 or 20 μg/mL) at 4°C for 1 h before infection. ERA-mCherry virus particles at an MOI of 0.1 were then added to the cells and incubated at 4°C for 1 h followed by three washes before further incubation in medium containing the corresponding antibodies at 37°C. At 48 h post-infection, the cell culture supernatant was collected for virus titer titration. The cells were then fixed with paraformaldehyde, and the infection ratio was determined using the PerkinElmer Operetta high-content system (PerkinElmer, Waltham, MA, USA). Fifty-two fields per well were imaged at 20× magnification. Columbus software (PerkinElmer, Waltham, MA, USA) was used to automatically identify and quantify green fluorescence and cell nuclei. The relative infection rate of the antibody-treated groups was calculated based on the normalized isotype control infection rate.

### Cell viability assay

Cell viability was assessed using the CellTiter-Glo kit (Promega, Madison, WI, USA). HEK293 or N2a cells were seeded onto 96-well plates with opaque walls. GRP78 monoclonal antibody or its isotype antibody, purified mouse IgM, was added at concentrations of 20 or 40 μg/mL. After 48 h, CellTiter-Glo reagent was added directly into each well and incubated with the cells for 10 min on a shaker to induce cell lysis. Luminescence was measured with a GloMax 96 Microplate Luminometer (Promega, Madison, WI, USA).

### Virus titration

For virus titration, BSR-T7/5 cells were seeded in 24-well plates and infected with 10-fold serial dilutions of the virus. After incubation for 48 h, focus-forming units in 24-well plates were counted, and the titer was expressed as the reciprocal of the highest dilution titer. We used the MOI determined in BSR-T7/5 cells as the standard relative dose.

### Viral entry assay

The internalization of ERA-mCherry in HEK293 cells and N2a cells was performed as previously described (34). Cells were transfected with the indicated siRNA for 24 h before being transferred onto ice for precooling for 20 min. ERA-mCherry (MOI = 1) then bound to the cells at 4°C for 1 h before being moved to 37°C for an additional hour to allow internalization. Subsequently, cells were washed three times with PBS and trypsin to remove surface-bound viruses before being lysed for total RNA extraction and subjected to qPCR to quantify internalized viruses.

### Isolation of cell plasma membrane proteins

To detect proteins on the cell membrane, HEK293 cells and N2a cells’ plasma membranes were extracted using the Minute Plasma Membrane Protein Isolation and Cell Fraction Kit (Invent Biotechnologies, SM-005). The isolated yields were then subjected to SDS-PAGE and Western blotting. The detection antibody used was against GRP78 (Abcam, ab108613), with Na/K ATPase (Abcam, ab76020) serving as the plasma membrane loading control.

### Plasmid construction

To construct expression vectors for the BiFC system, the C-terminal regions of GRP78, GRP78Δ19–50aa, RABV G, nAChR (GenBank accession no. NM_001039523.3), and mGluR2 (GenBank accession no. NM_000839.4) were fused in-frame with the N-terminal (residues 2–173) of the green fluorescent protein Venus, followed by a Flag tag. Similarly, the C-terminal of GRP78, GRP78Δ19–50aa, RABV G, NGFR (GenBank accession no. NM_002507.4), and NCAM (GenBank accession no. BC047244.1) were fused with the C-terminal 154 to 238 aa of Venus.

Plasmids encoding Flag-tagged NCAM, mGluR2, NGFR, GRP78, and ERA G protein (designated RVG; GenBank accession no. J02293.1) were constructed by inserting the respective coding sequences of NCAM, mGluR2, NGFR, GRP78, and ERA G into the pCAGGS vector, which drives the expression of recombinant proteins with a C-terminal Flag tag (DYKDDDDK). Plasmids expressing Myc-tagged RVG, NGFR, and nAChR were generated via the insertion of the corresponding coding sequences of RVG, NGFR, and nAChR into the same pCAGGS backbone, with the vector engineered to produce proteins carrying a C-terminal Myc tag (EQKLISEEDL). Additionally, plasmids encoding HA-tagged GRP78, GRP78Δ19–50aa, mGluR2, and nAChR were constructed by inserting the respective coding sequences of GRP78, GRP78Δ19–50aa, mGluR2, and nAChR into pCAGGS, which was modified to express target proteins fused with a C-terminal HA tag (YPYDVPDYA).

### Co-IP

HEK293 cells were transfected with plasmids using ExFect transfection reagent (Vazyme, T101-AA) following the manufacturer’s instructions. At 48 h post-transfection, cells were lysed with NP-40 lysis buffer for 1 h at 4°C. The supernatant was collected and mixed with 40 μL of protein A/G agarose (Abmart, A10001M) for 4 h at 4°C to remove nonspecific binding proteins. After being washed, the supernatant was mixed with anti-Flag antibody-conjugated agarose beads (Sigma, A2220) for 6 h at 4°C. The beads were isolated by centrifugation, washed five times with NP-40 lysis buffer, and used for SDS-PAGE and Western blotting.

### IFA

For immunofluorescence assay, cells were fixed with paraformaldehyde and 0.1% Triton for 10 min at room temperature to permeabilize the cells. After incubating with PBS containing 5% BSA for 1 h at room temperature to block nonspecific protein-protein interactions, the cells were incubated with primary antibodies against Flag or Myc tags at a 1:200 dilution in PBS with 5% BSA for 2 h. After five washes with PBS, the cells were stained with TRITC-conjugated secondary antibodies (ZSBio, ZF-0316) at a 1:200 dilution for 1 h. Subsequently, the cells underwent five additional washes with PBS and were then incubated with DAPI (Sigma-Aldrich, D9542) for 10 min to facilitate nuclear staining. Following further washing with PBS, the cells were observed under a confocal microscope.

### Statistical analysis

The analyses were conducted using GraphPad Prism v.8.0.2.

## Data Availability

The data supporting the findings of this study are available within the article.

## SUPPLEMENTAL MATERIAL

Fig. S1 jvi.00039-26-s0001.pdf Viability of GRP78-silenced HEK293 cells and N2a cells.

Fig. S2 jvi.00039-26-s0002.pdf Viability of antibody-treated HEK293 cells and N2a cells.

ASM does not own the copyrights to Supplemental Material that may be linked to, or accessed through, an article. The authors have granted ASM a non-exclusive, world-wide license to publish the Supplemental Material files. Please contact the corresponding author directly for reuse.
