# Supplementary material for: GRP78 promotes rabies virus entry through interacting with viral receptors
Source: J Virol. 2026 Apr 14;100(5):e00039-26. doi: 10.1128/jvi.00039-26 (PMC13185585; doi:10.1128/jvi.00039-26)
Supplement: Fig. S1 — Viability of GRP78-silenced HEK293 cells and N2a cells. [file jvi.00039-26-s0001.pdf]

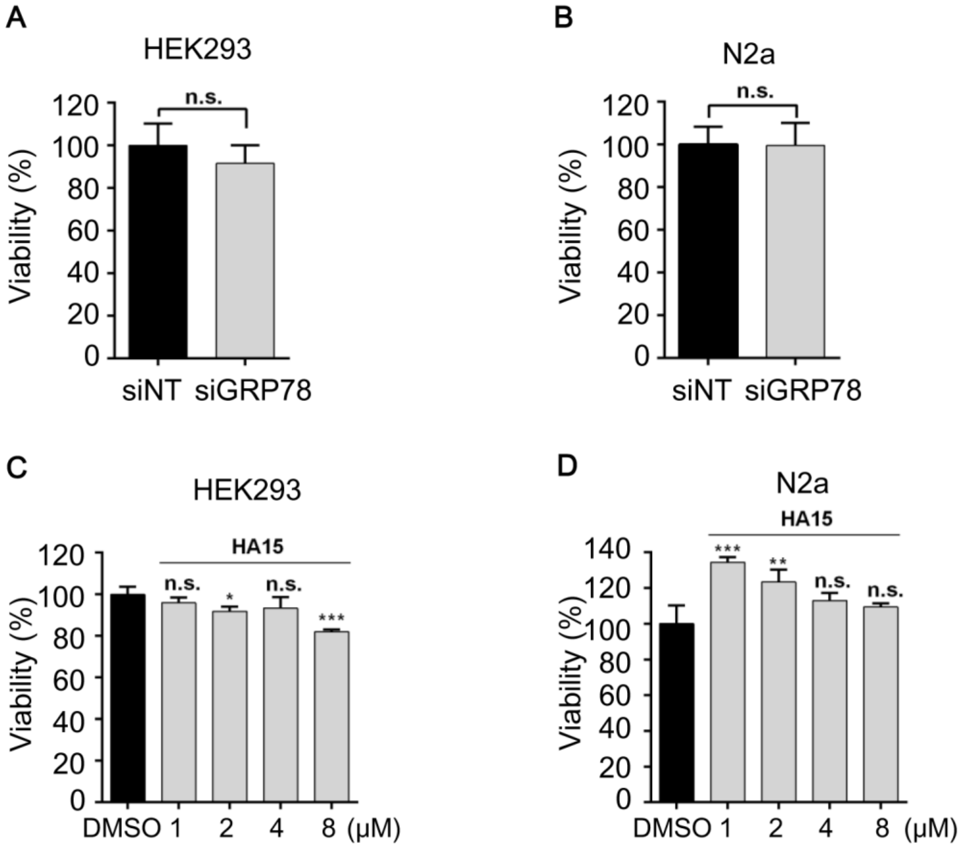

Fig S1. The viability of GRP78-silenced HEK293 cells and N2a cells. The viability of GRP78-silenced HEK293 cells (A) and N2a cells (B) were determined by using the Cell Titer Glo kit at 72 hours post-transfection. Data are relative fluorescence values normalized to the negative control. The viability of HA15 treated HEK293 cells (C) and N2a cells (D) were determined at 48 hours post-treated. Data are relative fluorescence values normalized to the DMSO control. All data represent the mean ± SD from three repeats. A one-way ANOVA was used for the statistical analysis. *, $p<0.05$,**, $p < 0.01$, ***$p < 0.001$; n.s., not significant.
