# Supplementary material for: GRP78 promotes rabies virus entry through interacting with viral receptors
Source: J Virol. 2026 Apr 14;100(5):e00039-26. doi: 10.1128/jvi.00039-26 (PMC13185585; doi:10.1128/jvi.00039-26)
Supplement: Fig. S2 — Viability of antibody-treated HEK293 cells and N2a cells. [file jvi.00039-26-s0002.pdf]

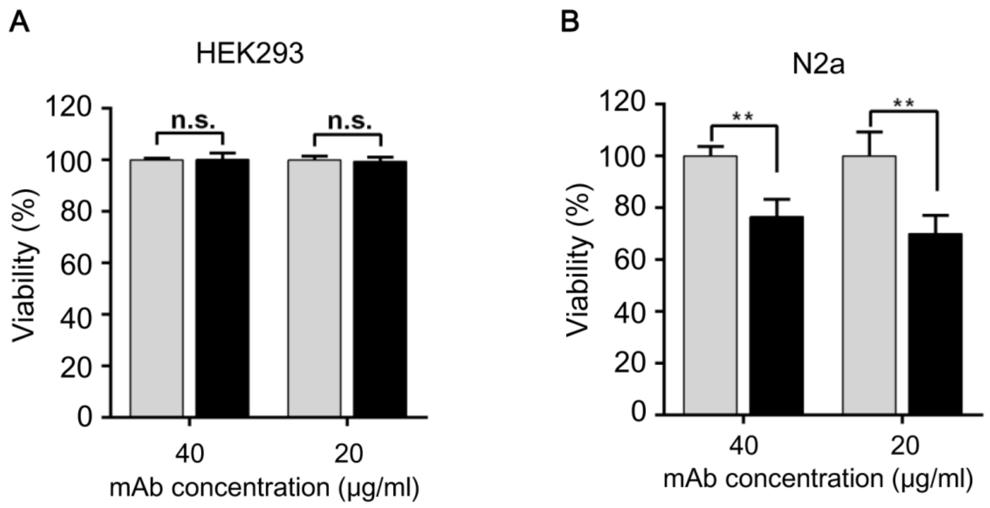

Fig S2. The viability of antibodies treated HEK293 cells and N2a cells. (A and B) The viability of mAb or isotype IgM treated HEK293 cells (A) and N2a cells (B) at 48 hours post-treated were determined by using the Cell Titer Glo kit. Values represent the mean ± SD. A two-tailed unpaired Student's *t*-test was used for the statistical analysis. ***p < 0.01*; n.s., not significant.
